# Type 17 Immune Response Facilitates Progression of Inflammation and Correlates with Cognition in Stable Schizophrenia

**DOI:** 10.3390/diagnostics10110926

**Published:** 2020-11-10

**Authors:** Milica M. Borovcanin, Slavica Minic Janicijevic, Ivan P. Jovanovic, Nevena M. Gajovic, Milena M. Jurisevic, Nebojsa N. Arsenijevic

**Affiliations:** 1 Department of Psychiatry, Faculty of Medical Sciences, University of Kragujevac, Kragujevac 34 000, Serbia; 2 PhD Studies, Faculty of Medical Sciences, University of Kragujevac, Kragujevac 34 000, Serbia; redslavica@yahoo.com; 3 Center for Molecular Medicine and Stem Cell Research, Faculty of Medical Sciences, University of Kragujevac, Kragujevac 34 000, Serbia; ivanjovanovic77@gmail.com (I.P.J.); gajovicnevena@yahoo.com (N.M.G.); arne@medf.kg.ac.rs (N.N.A.); 4 Department of Clinical Pharmacy, Faculty of Medical Sciences, University of Kragujevac, Kragujevac 34 000, Serbia; milena.jurisevic13@gmail.com

**Keywords:** schizophrenia, cognition, IL-17, CD4^+^ T cells, CD56^+^ NK cells

## Abstract

Dysregulation of the type 17 immune pathway has already been considered in schizophrenia and we previously measured decreased sera values of interleukin (IL)-17 in early stages. We further explored the possible correlation of IL-17 systemic levels with proinflammatory cytokines and cognitive scores and additionally analyzed the percentage of IL-17 producing lymphocytes in peripheral blood of patients with stable schizophrenia. We included 27 patients diagnosed with schizophrenia (F20), after a three-month stable depot antipsychotic therapy (risperidone or paliperidone) and 18 healthy control subjects. Positive and Negative Syndrome Scale of Schizophrenia and the Montreal-Cognitive Assessment (MoCA) were conducted. Sera concentrations of IL-17, IL-6, tumor necrosis factor alpha (TNF-α) and soluble ST2 receptor (sST2) were measured. Flow cytometry and Natural Killer (NK) and T cell analyses were done in 10 patients and 10 healthy controls. Moderate positive correlation was established between IL-17 and TNF-α (r = 0.640; *p* = 0.001), IL-17 and IL-6 (r = 0.514; *p* = 0.006), IL-17 and sST2 (r = 0.394; *p* = 0.042). Furthermore, a positive correlation between the serum levels of IL-17 and MoCA scores was observed, especially with visuospatial and executive functioning, as well as language functioning and delayed recall (*p* < 0.05). Significantly higher percentage of IL-17 producing CD56^+^ NK cells was measured in peripheral blood of patients with schizophrenia in remission vs. healthy individuals (*p* = 0.001). The percentage of CD4^+^ T cells and CD4^+^ T cells that produce IL-17 was significantly increased in patients (*p* = 0.001). This study revealed the involvement of innate type 17 immune response in the progression of inflammation and this could be related to cognitive functioning in stable schizophrenia.

## 1. Introduction

In the last half of the century, many biomarker candidates in mental disorders have been described. They could be applicable as a trait marker in establishing diagnosis, as a state marker in exacerbation or even in predicting the treatment response and outcome [1]. The observation that some immunological pathways have been especially active was a basis for developing new therapeutic strategies.

Our research group suggested that transforming growth factor-beta (TGF-β) could be a trait marker for distinguishing patients with psychosis (sensitivity of 70.4% and specificity of 80.6%) [2]. Further, we recently measured elevated sera levels of galectin-3 in remission and interleukin (IL)-33 in schizophrenia exacerbation, biomarkers that are used as predictors of all-cause mortality in acute and chronic heart failure [3]. The heterogeneous data about state and trait markers could reflect the core problem of the schizophrenia concept, representing a combination of syndromes rather than a single clinical entity. Some authors suggested that it could be useful to subdivide this population of patients with schizophrenia into more homogenous subgroups for cytokine measuring and study combinations of biomarkers, including immune signatures [4].

The evolution of the immune system has enabled a highly differentiated immune defense of the organism, within two functionally different immune systems, innate and acquired [5]. There are two main mechanisms of the acquired immune system – humoral, which is mediated by B cells, and cellular, which is mediated by CD4^+^ and CD8^+^ T cells. Through the response to different antigens, CD4^+^ T cells can be differentiated into Th1, Th2 or Th17 subpopulations, based on the unique cytokines that they produce [6]. Recently, innate cells have been further subdivided into type 1, type 2 and type 3 innate lymphoid cells, according to the produced cytokine sets [7]. Type 1 immunity is mediated by interferon gamma (IFN-γ)—producing innate lymphoid cells type 1, CD8^+^ cytotoxic T cells and CD4^+^ Th1 cells. Type 2 immunity refers to innate lymphoid cells type 2 and Th2 cells that produce IL-4, IL-5 and IL-13. Type 3 immunity consists of innate lymphoid cells type 3 and Th17 cells producing IL-17 and IL-22 [7]. Taken altogether, the innate as well as acquired immune system can be subdivided into three types of immune response, based on the produced cytokines: type 1, type 2 and type 3/17.

Several studies examined the importance of innate and acquired immune system cells in patients with schizophrenia. Different examinations focused on acquired immunity showed the predomination of type 1 cytokines, while other studies claimed increased Th2 cells in the blood of patients with schizophrenia [8,9]. Lower numbers of Natural Killer (NK) cells, as a component of the innate immune system characterized by the expression of the phenotypic marker CD56^+^, have been detected in drug-naïve and medicated patients with schizophrenia compared to healthy controls [10]. In the other study that included nonmedicated schizophrenia patients, differences in NK cell count were not observed, whereas increased activity per NK cell was registered [11].

Various studies have already confirmed the importance of IL-17 in animal models of autoimmunity and human autoimmune diseases, such as multiple sclerosis, rheumatoid arthritis and psoriasis [12]. Moreover, the interconnection between IL-17 and the pathogenesis of various mental disorders was also detected. The elevated levels of IL-17 in amyloid-β (Aβ) 1-42-induced Alzheimer’s dementia (AD) brain suggests that peripheral Th17 cells can penetrate the brain and mediate AD neuroinflammation and in this way have a role in the pathogenesis of AD [13]. Some reports indicated increased serum levels of IL-17 in individuals with autism spectrum disorder (ASD) [14,15], but other studies indicated no statistically significant differences in levels of IL-17 in plasma or serum [16,17]. Divanni et al. (2016) [18] have shown that the level of IL-17 in the patient group with major depressive disorder (MDD) was significantly higher than that of the healthy controls. Moreover, IL-17A levels were significantly higher in patients with obsessive-compulsive disorder (OCD) vs. healthy control group [19].

Dysregulation of the IL-17 pathway in schizophrenia has been suggested by Subbanna and colleagues (2018) [20]. Our research group previously published results suggesting lower IL-17 sera concentrations in drug-naïve patients with first episode of psychosis [2]. There is also evidence of the activation of Th17 cells in patients with first episode schizophrenia [21], and Himmerich et al. (2011) [22] demonstrated in vitro that antipsychotics could increase IL-17 production. Considering the possible correlation with psychopathology, we already established a correlation between IL-17/TGF-β ratio and negative and general psychopathology subscales [2]. According to our knowledge, the connection of type 17 immune response with representatives of systemic inflammation and cognitive functioning has not been thoroughly investigated in patients with stable schizophrenia. Additionally, we analyzed the percentage of IL-17-producing lymphocytes in the peripheral blood of patients with stable schizophrenia.

## 2. Materials and Methods

### 2.1. Subjects

A total of 27 patients with schizophrenia in remission after a three-month stable depot antipsychotic therapy of risperidone or paliperidone and 18 healthy control subjects were included in this study. The study was approved by the Ethics Committee of Clinical Centre Kragujevac (number 01-7015, date 2 July 2015) and was conducted in compliance with the ethical principles. Informed consent had been obtained from all patients before any study procedures began. Our university psychiatric clinic provides settings for the application of antipsychotic depot formulations (risperidone and paliperidone) in the Psychiatric Daily Hospital. All these patients have been considered and some of them selected and included in this study. Diagnosis was established using International Statistical Classification of Diseases and Related Health Problems, Tenth Revision (ICD-10) [23], F20 for the schizophrenia in remission group. In the diagnostic algorithm for schizophrenia, we were obliged to exclude organic conditions of the central nervous system (neurological examination, using computerized tomography, magnetic resonance and electroencephalography, etc.). The diagnosis of schizophrenia that was already previously established has been confirmed before involving patients in the study. The exclusion criteria were any comorbidities that could have influenced the results, especially current infections, allergies or autoimmune disorders, or current anti-inflammatory or antiviral medications. Neither psychotic patients nor controls suffered from substance or alcohol abuse, nor were any comorbidities of other mental illnesses diagnosed. Healthy controls were recruited through the process of blood donation at the Service Supply of Blood and Blood Products, Clinical Center Kragujevac.

### 2.2. Psychological Assessment

Psychopathology was evaluated using the Positive and Negative Syndrome Scale of Schizophrenia (PANSS) consisting of positive, negative and general psychopathology subscale, and confirmed criteria for remission [24]. Montreal-Cognitive Assessment (MoCA) was used to assess cognitive functioning, including domains of attention, concentration, executive functions, memory, language, visuoconstructive skills, conceptualization and orientation [25].

### 2.3. Measurements of Cytokine Levels in Sera

The blood was obtained from patients and control individuals in the morning (approximately 8 a.m.), and sera were separated, collected and stored at −80 °C before use. Concentrations of tumor necrosis factor alpha (TNF-α), IL-6, IL-17 and sST2 were measured in the serum of schizophrenia patients and control subjects by using commercially available enzyme-linked immunosorbent assay (ELISA) tests, according to the manufacturer’s instructions (R&D Systems, Minneapolis, Minnesota, USA). Values are presented as pg/mL.

### 2.4. Flow Cytometric Analysis of Peripheral Blood NK and T Cell

Immune cells were isolated from peripheral blood (peripheral blood mononuclear cells—PBMCs) of 10 patients with SC in remission and 10 HC, as previously described [26]. PBMCs were prepared using Histopaque (Sigma Aldrich Company Ltd., Saint Louis, MO, USA) density gradient centrifugation for 20 min at 690 g. After centrifugation, the interphase was carefully removed and single-cell suspensions were washed twice with fluorescence-activated cell sorter (FACS) Dulbecco‘s modified eagle medium (DMEM) containing 10% fetal bovine serum (Gibco, USA). For flow cytometry, isolated PBMCs were incubated with anti-human CD4 (S3.5), CD56 (39D5) or isotype matched control conjugated with fluorescein isothiocyanate (FITC; BD Biosciences, Franklin Lakes, NJ, USA). Evaluation of intracellular expression of IL-17 and IL-10 in NK and T cells derived from peripheral blood was carried out as previously described in detail [26]. Cells were stimulated using phorbol 12-myristate 13-acetate (50 ng/mL, Sigma-Aldrich Company Ltd., Saint Louis, MO, USA), ionomycin (500 ng/mL, Sigma-Aldrich Company Ltd., Saint Louis, MO, USA) and GolgyStop (BD Pharmingen, NJ, USA) for 4 hours at 37 °C, 5% CO2. After fixation and permeabilization with Cytofix/Cytoperm, cells were permeated with 0.1% saponin and stained with fluorochrome-labeled anti-human mAbs specific for IL-17 (CZ8-23G1) and IL-10 (JES3-9D7). Flow cytometry was conducted on FACSCalibur flow cytometer (BD Biosciences, San Jose, USA). The data were analyzed using FlowJo (Tree Star) version 10.

### 2.5. Statistical Analysis

All statistical analyses were performed using IBM SPSS Statistics for Windows, version 23 (IBM Corp., Armonk, NY, USA). The numeric data were given as mean ± standard deviation or standard error. The normality of data distribution was tested by Shapiro–Wilk test. Data were analyzed by Student’s t-test or Mann–Whitney U-test as appropriate. The possible relationship between cytokine levels and MoCA scores/PANSS scores with stable schizophrenia was evaluated by Pearson’s or Spearman’s correlation as appropriate. A *p*-value < 0.05 was considered to be statistically significant.

## 3. Results

### 3.1. Demographic and Clinical Characteristics

A total of 27 patients (schizophrenia in remission—SC in remission) and 18 healthy control subjects (healthy controls—HC) were included in this study and demographic and clinical characteristics of the sample were previously presented in Borovcanin et al. (2018) [3] and are shown in Table 1.

In brief, the comparison of patients and control subjects did not reveal any statistically significant differences in age or gender. The duration of illness was 9.95 ± 7.71 years, with two hospitalizations on average (2.18 ± 1.92). Patients were treated with depot formulations of risperidone and paliperidone (22 vs. 5 patients). Overall psychopathology was assessed as: PANSS total score (99.22 ± 18.23), positive syndrome scale (22.26 ± 5.97), negative syndrome scale (27.52 ± 6.09) and general psychopathology scale (49.44 ± 7.83). MoCA total score and subscores used in the schizophrenia group were as follows: MoCA total score 22.74 ± 4.76; visuospatial/executive 4.11 ± 1.25; naming 2.78 ± 0.69; attention 5.07 ± 1.21; language 1.89 ± 0.69; abstraction 1.41 ± 0.84; delayed recall 1.81 ± 1.62; orientation 5.74 ± 0.81.

The cytokine levels that were measured are presented in Table 2. Systemic values of TNF-α were significantly higher in patients with schizophrenia (*p* =0.030), while there was no difference in the concentrations of IL-17, IL-6 and sST2 between patients with SC in remission and healthy controls, although we noticed the same trend as for TNF-α (Table 2).

### 3.2. Systemic Level of Il-17 Positively Correlated with Proinflammatory Mediators

The relationship between serum values of IL-17 and proinflammatory soluble factors was explored. The analysis revealed a positive correlation between serum IL-17 and proinflammatory mediators TNF-α, IL-6 and sST2 in patients with schizophrenia and the results are summarized in Table 3. The strength of correlation was determined using Spearman’s test. There is a moderate positive correlation between IL-17 and TNF-α (r = 0.640; *p* = 0.001), IL-17 and IL-6 (r = 0.514; *p* = 0.006), IL-17 and sST2 (*r* = 0.394; *p* = 0.042).

### 3.3. Systemic Level of Il-17 Positively Correlated with Cognitive Scoring

**Figure 1:**
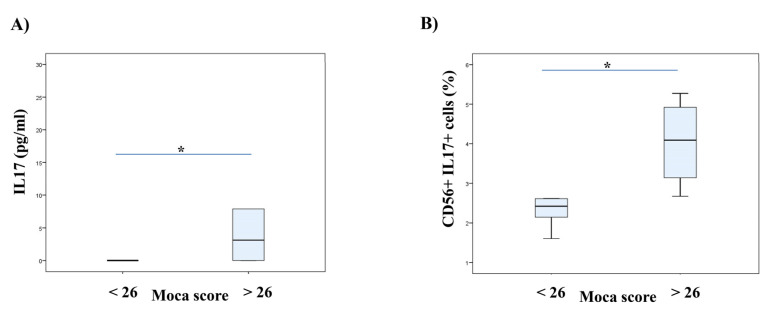
Serum concentrations of IL-17 and the percentage of NK cells secreting IL-17 regarding cognition of patients with stable schizophrenia. Patients with stable schizophrenia in remission were divided into two groups based on MoCA scores (score 26 as a cut-off value). The serum levels of IL-17 were determined by ELISA and IL-17 production of NK cells was examined by flow cytometry. The Student’s t or Mann–Whitney U test was applied to evaluate statistically significant differences between two groups, * *p* < 0.05. In patients with higher MoCA scores were detected higher serum concentrations of IL-17 (**A**) and also the higher percentage of IL-17 secreting CD56^+^ NK cells (**B**). MoCA—Montreal-Cognitive Assessment; NK–Natural Killer Cells.

We established a positive correlation between the serum levels of IL-17 and MoCA scores in patients with SC in remission. There was no correlation between the serum levels of IL-17 and PANSS scores in the examined patients. However, a moderate negative correlation was detected between the percentage of IL-17 producing CD56^+^ NK cells and G16 item score that refers to social avoidance (r = −0.649; *p* = 0.042), and a moderate positive correlation with total MoCA score (*r* = 0.650; *p* = 0.042) and a strong positive correlation with visuospatial/executive function score were observed (r = 0.817; *p* = 0.004). An examination of the possible connection between IL-17 sera concentration and MoCA scores revealed the following results: significant weak positive correlation with total score (*r* = 0.387; *p* = 0.046), and moderate with language (fluency) (*r* = 0.569; *p* = 0.002) and delayed recall (*r* = 0.416; *p* = 0.031) scores. We divided patients according to MoCA score: 1) MoCA < 26 and 2) MoCA > 26. We detected higher serum concentrations of IL-17 and the percentage of IL-17 secreting CD56^+^ NK cells in patients with higher MoCA scores (MoCA > 26), as presented in Figure 1.

### 3.4. Higher IL-17 Expression on Peripheral Blood NK cells in Stable Phase of Schizophrenia

**Figure 2:**
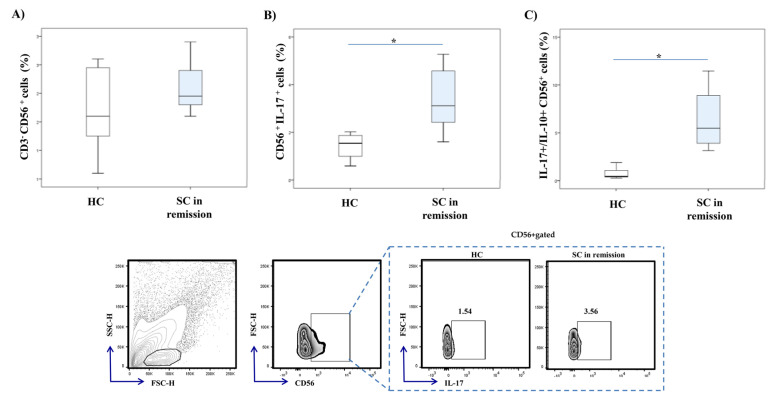
IL-17 and IL-10 production in peripheral blood NK cells. Cytokine production of peripheral blood NK cells was examined by flow cytometry. The graph and representative FACS plots display the percentage of IL-17 producing CD56^+^ cells and IL-17^+^/IL-10^+^ CD56^+^ cells ratio in peripheral blood of patients with stable schizophrenia and healthy controls, respectively. The Student’s t or Mann–Whitney U test was applied to evaluate statistically significant differences between two groups, * *p* < 0.05. There were no significant differences in the percentage of CD56^+^ NK cells between patients with SC in remission and HC (**A**). Significantly higher percentage of IL-17 producing CD56^+^ NK cells was observed in patients with SC in remission (**B**). The ratio of IL-17 and IL-10 expression on NK cells was significantly higher in patients (**C**). SC in remission—schizophrenia in remission; HC—healthy controls; FACS–fluorescence-activated cell sorter.

We have examined CD56^+^ NK cells in peripheral blood of patients with stable schizophrenia. We did not find any significant differences in the percentage of CD56^+^ NK cells between patients with stable schizophrenia and HC (Figure 2A). However, intracellular staining of NK cells revealed a significantly higher percentage of IL-17 producing CD56^+^ NK cells (*p* = 0.001) in patients with SC in remission (Figure 2B). Moreover, the ratio of IL-17 and IL-10 expression on NK cells was significantly higher in patients (*p* = 0.001; Figure 2C).

### 3.5. Accumulation of CD4^+^ T Cells and Higher Il-17 Expression on Peripheral Blood CD4^+^ T Cells in Stable Phase of Schizophrenia

**Figure 3:**
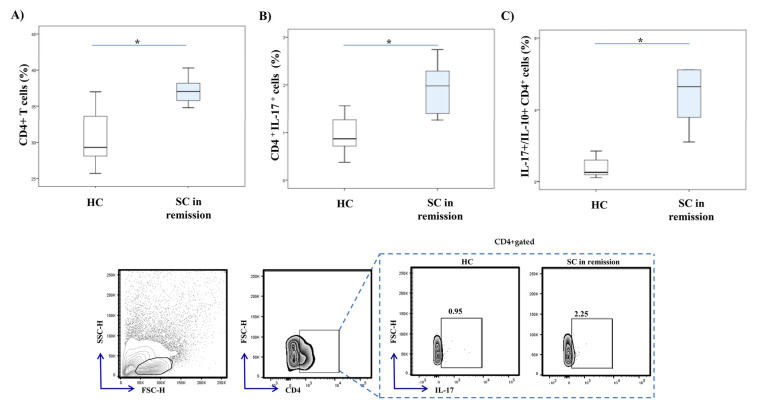
IL-17 and IL-10 production in peripheral blood CD4^+^ Th cells. Percentages and cytokine production of peripheral blood CD4^+^ cells were examined by flow cytometry. The graph and representative FACS plots display the percentage of CD4^+^ cells, IL-17 producing CD4^+^ cells and IL-17^+^/IL-10^+^ CD4^+^ cells ratio in peripheral blood of patients with SC in remission and healthy controls, respectively. The Student’s t or Mann–Whitney U test was applied to evaluate statistically significant differences between two groups, * *p* < 0.05. Higher percentage of CD4^+^ T cells was measured in the peripheral blood of SC in remission patients in comparison to healthy individuals (**A**). The percentage of CD4^+^ T cells that produce IL-17 was significantly increased in SC in remission vs. HC group (**B**). The ratio of IL-17 and IL-10 producing CD4^+^ T cells was significantly higher in patients with SC in remission (**C**). SC in remission—schizophrenia in remission; HC—healthy controls; FACS–fluorescence-activated cell sorter.

Flow cytometric analysis revealed a significantly higher percentage of CD4^+^ T cells (*p* = 0.001) in the peripheral blood of SC in remission patients in comparison to healthy individuals (Figure 3A). The percentage of CD4^+^ T cells that produce IL-17 was significantly increased in SC in remission vs. HC group (*p* = 0.001; Figure 3B). Additionally, the ratio of IL-17 and IL-10 producing CD4^+^ T cells was significantly higher in patients with SC in remission (*p* = 0.001; Figure 3C).

Age, sex, BMI and dosage of antipsychotics did not affect the systemic level of mediators of interest or percentage of PBMCs subpopulations and IL-17 expression in PBMCs. There was no significant difference in these parameters among patients divided into groups according to age, sex, BMI and dosage of antipsychotics, respectively, as there was no correlation between these parameters and age, sex, BMI and dosage of antipsychotics (data not shown).

## 4. Discussion

The type 17 immune response characteristic is IL-17, a potent mediator of the inflammatory response in autoimmune diseases [27,28]. In the most recent animal study of Tfilin and Turgeman (2019) [29], it was suggested that IL-17 could regulate adult neurogenesis in two different aspects: by inhibiting neuroprogenitor proliferation and promoting the maturation of already formed neuroblasts. Previous studies claimed that there is a linkage between IL-17 and inflammation based-neurological diseases. It has been speculated that IL-17 affects tight junctions between endothelial cells in the blood–brain barrier, thus allowing Th17 cells, one of the main sources of IL-17, to enter easily and accumulate in the central nervous system (CNS) [30]. The other possible mechanism is that Th17 cells enter easily through the choroid plexus in the CNS by constitutively expressing chemokine receptor CCR6, which interacts with CCL20 on epithelial cells of the choroid plexus [31]. IL-17 is expressed in some pathophysiological conditions, such as ischemic brain lesion [32]. The fact that mild localized perivascular inflammation was also identified in schizophrenia is of particular interest [33].

In light of the above considerations, it has been shown that IL-17 is also involved in the pathogenesis of various mental disorders. We already mentioned the potential role of the IL-17 pathway in the etiology of schizophrenia, but the inflammatory hypothesis of AD, ASD, MDD and OCD provides further support for the Th17-mediated pathogenesis of schizophrenia. The exact role of IL-17 in the genesis and progression of schizophrenia is not still clear. A higher percentage of Th17 cells and higher values of IL-17 in patients with first-episode and drug-naive schizophrenia were suggested by Ding et al. (2014) [21]. The elevation of IL-17 in schizophrenia could be a consequence of transforming growth factor beta (TGF-β)-stimulated differentiation by IL-23, both cytokines that were shown to be elevated through schizophrenia continuance [34,35]. In the recent meta-analysis, there were no specific changes regarding levels of IL-17 in first-episode psychosis patients and the authors even discussed that IL-17 may not be involved in the pathophysiology of schizophrenia [36]. Interestingly, our previous study showed decreased values of IL-17 in first-episode and non-treated schizophrenia subjects [2]. Similar to our results, reduced levels of IL-17F were measured in drug-naїve schizophrenia patients [20], while others have published negative results [37]. Here, we revealed a slight increment of IL-17 concentration in the sera of patients with SC in remission, in comparison to healthy control subjects (Table 2).

Further, we analyzed the percentage of IL-17 producing lymphocytes in peripheral blood. Significantly higher percentages of IL-17 producing CD56^+^ NK cells and CD4^+^ Th lymphocytes, respectively, were detected in peripheral blood of patients with SC in remission, in comparison to healthy controls (Figure 2 and Figure 3). For the first time, our data revealed the involvement of innate, besides acquired, type 17 immune response in the pathogenesis of schizophrenia. Furthermore, the ratio of IL-17 and IL-10 expression in NK cells and CD4^+^ T cells was significantly increased in patients with stable schizophrenia (Figure 2 and Figure 3). These results are in line with the previous study showing a decreased level of anti-inflammatory IL-10 cytokine in patients with first-episode schizophrenia [38]. Altogether, the presented data emphasize the predomination of proinflammatory IL-17 over anti-inflammatory IL-10 production in innate and acquired lymphocytes derived from peripheral blood of SC in remission patients.

In this study, we also analyzed the relation between serum values of IL-17 and proinflammatory mediators of interest. Our results revealed a moderate positive correlation between systemic IL-17 and IL-6, TNF-α and sST2 (Table 3). Recent studies confirmed that the main target of the IL-17 signaling pathway is NFκB transcriptional factor, which is known to be involved in the inflammatory response [39,40]. Moreover, evidence suggests that there is a linkage between IL-17 and other proinflammatory cytokines. It has been reported that by binding to its receptor, IL-17 stimulates the production of IL-1β, IL-6 and TNF-α that further facilitates the enlargement of the inflammatory process [41]. As the sST2 molecule is a decoy receptor for cytokine IL-33, thus blocking IL-33/ST2 signaling, our previous results showing enhanced systemic IL-17 in mice without IL-33/ST2 signaling are in line with the present findings [42,43]. The presented data confirm the stimulating effect of IL-17 on ongoing inflammation in patients with schizophrenia in remission.

Vergaelen and colleagues (2018) [44] discussed that the defects in the T-regulatory system allow a proinflammatory state in response to normal environmental cues in patients with schizophrenia spectrum disorder and the significant increase in Th17 cell circulating levels was related to psychotic symptoms in patients with 22q11.2 deletion syndrome. A proinflammatory profile was suggested in patients with ultra-high risk of developing psychosis, because decreased levels of IL-17 were measured, and a positive correlation was established with global assessment of functioning scale total scores [45]. Elevated plasma levels of IL-17, TGF-β and IL-23, cytokines that belong to the Th17 pathway, were measured in patients with schizophrenia exacerbation and at the same time correlated with severity of illness and aggressive behavior [46]. Bizarre behavior and apathy could relate to IL-17A driven inflammation in a specific subset of patients with schizophrenia [20].

Finally, we investigated the relation between IL-17 and cognitive impairment in patients with schizophrenia (Figure 1). According to the results of this study, IL-17 producing CD56^+^ NK cells could have a beneficial role in improving social functioning. Our results also revealed a positive correlation between serum levels of IL-17 and MoCA scores in the examined patients, suggesting its moderate impact on global cognitive functioning and strong influence on visuospatial and executive functioning, as well as language functioning and delayed recall. Antipsychotic-naive/free schizophrenia patients showed significantly lower IL-17 levels and a significant negative correlation with dimension characterized by inattention, formal thought disorder and alogia [47]. Anti-IL-17A may represent a new therapeutic strategy for the treatment of endotoxemia-induced neuroinflammation and cognitive dysfunction in aged rats [48]. Together with our results of the association of IL-17 with cognitive impairment in schizophrenia, this can give an impetus for further research in this direction, for a better understanding of the schizophrenia etiopathogenesis, as well as the improvement and preservation of cognition.

The possible impact of antipsychotics could not be ruled out, especially considering their beneficial effects in improving cognitive functioning and even adult hippocampal neurogenesis [49,50,51]. In Iranian patients with schizophrenia in remission that were treated with clozapine, no significant difference in IL-17 levels was observed [52]. Moreover, in the meta-analysis of Capuzzi et al. (2017) [53], the significant variation in plasma levels pre- and post-antipsychotic treatment was not detected. Neuroleptic-free patients in the acute phase of disease exhibited higher IL-17 serum levels, so the authors of this study consider that this elevation is not due to antipsychotic treatment, but rather related to the intrinsic profile of the disease [54]. Our results must be interpreted in the context of the phase of illness and the idea that these effects could be specific for this antipsychotic regimen. Consequently, the subgrouping of this heterogenous population should be considered in the design of further research.

Although Th17 cells were shown to have significant implications in schizophrenia, they were predominantly involved in mucosal defense of lungs, skin and gut [55]. Debnath and Berk (2014) [56] have discussed the impact of complement activation and the altered gut microbiota on this mediator. It was suggested that add-on treatment with probiotics, and not standard antipsychotic treatment, exerted their effect through IL-17-related immune response, improving bowel function and preserving intestinal epithelium integrity [57].

A possible limitation of this study could be that anti-N-methyl-D-aspartate receptor encephalitis was not ruled out, but clinical presentation and therapeutic response have not pointed in this direction. The limitation of our work is that this was a cross-sectional study with only one time point evaluation, small sample size and also the fact that we cannot exclude the potential impact of some additional confounding factors.

## 5. Conclusions

This study reveals, for the first time, the involvement of innate type 17 immune response, besides acquired, as well as the predomination of proinflammatory IL-17 over anti-inflammatory IL-10 in the peripheral blood of patients with schizophrenia in remission. Additionally, the predomination of IL-17 facilitates the progression of inflammation, manifested by a positive correlation of systemic IL-17 and proinflammatory mediators IL-6, TNF-α and sST2. Presented data indicate that IL-17 could be therapeutically targeted to preserve cognitive potential, but these actions must be balanced because these changes could also be related to the manifestation of psychotic symptoms or aggressive behavior.

## Figures and Tables

**Table 1:** Demographic and clinical characteristics.

	SC in Remission (*n* = 27)	HC (*n* = 18)	*p*
Age (years mean ± SD)	36.18 ± 9.27	37.66 ± 9.96	0.886
Sex (male/female)	16/11	12/6	0.851
Duration of illness (years mean ± SD)	9.95 ± 7.71		
Number of previous hospitalizations	2.18 ± 1.92		
PANSS total score	99.22 ± 18.24		
MoCA total score	22.74 ± 4.76		
BMI	25.38 ± 5.38		

SD—standard deviation; SC in remission—schizophrenia in remission; HC—healthy controls; PANSS—Positive and Negative Syndrome Scale of Schizophrenia; MoCA—Montreal-Cognitive Assessment; BMI—body mass index.

**Table 2:** Cytokine concentrations (pg/mL; mean ± SD).

	SC in Remission (*n* =27)	HC (*n* =18)	*p*
**IL-17**	1.99 ±5.87	1.39 ±2.71	0.605
**IL-6**	15.76 ± 38.58	13.56 ± 3.10	0.232
**sST2**	936.03 ± 347.20	867.77 ± 407.44	0.747
**TNF-α**	21.35 ± 42.71 *	8.01 ± 1.68	0.030

SD—standard deviation; SC in remission—schizophrenia in remission; HC—healthy controls. * *p* < 0.05.

**Table 3:** Positive correlation of IL-17 with TNF-α, IL-6 and sST2 in sera of patients.

Serum Concentration (pg/mL)	IL-17	
	Spearman’s rho	*p* Value
**IL-6**	0.514	0.006
**TNF-α**	0.640	0.006
**sST2**	0.394	0.042
